# Supplementary material for: Reviving the sound of a 150-year-old insect: The bioacoustics of Prophalangopsis obscura (Ensifera: Hagloidea)
Source: PLoS One. 2022 Aug 10;17(8):e0270498. doi: 10.1371/journal.pone.0270498 (PMC9365155; doi:10.1371/journal.pone.0270498)
Supplement: S1 Table — (DOCX) [file pone.0270498.s001.docx]

Supporting Information for:

Reviving the sound of a 150-year-old Insect: the bioacoustics of *Prophalangopsis obscura* (Ensifera: Hagloidea)

Charlie Woodrow^1^, Ed Baker^2^, Thorin Jonsson^3^ & Fernando Montealegre-Z^1,4^

**S1 Table**

| Measurement | Left stridulatory file | Right stridulatory file |
| --- | --- | --- |
| Inter-tooth distance (Mean ± SD) | 72.87 ± 10.44 µm | 71.66 ± 8.02 µm |
| Tooth length | 92.30 ± 15.46 µm | 97.31 ± 17.66 µm |
| File length | 9.60 | 9.99 |
| Number of teeth | 134 | 137 |

Morphological characters of the tegmina stridulatory files of *P. obscura*.
