# Supplementary figures and images for: Reviving the sound of a 150-year-old insect: The bioacoustics of Prophalangopsis obscura (Ensifera: Hagloidea)
Source: PLoS One. 2022 Aug 10;17(8):e0270498. doi: 10.1371/journal.pone.0270498 (PMC9365155; doi:10.1371/journal.pone.0270498)

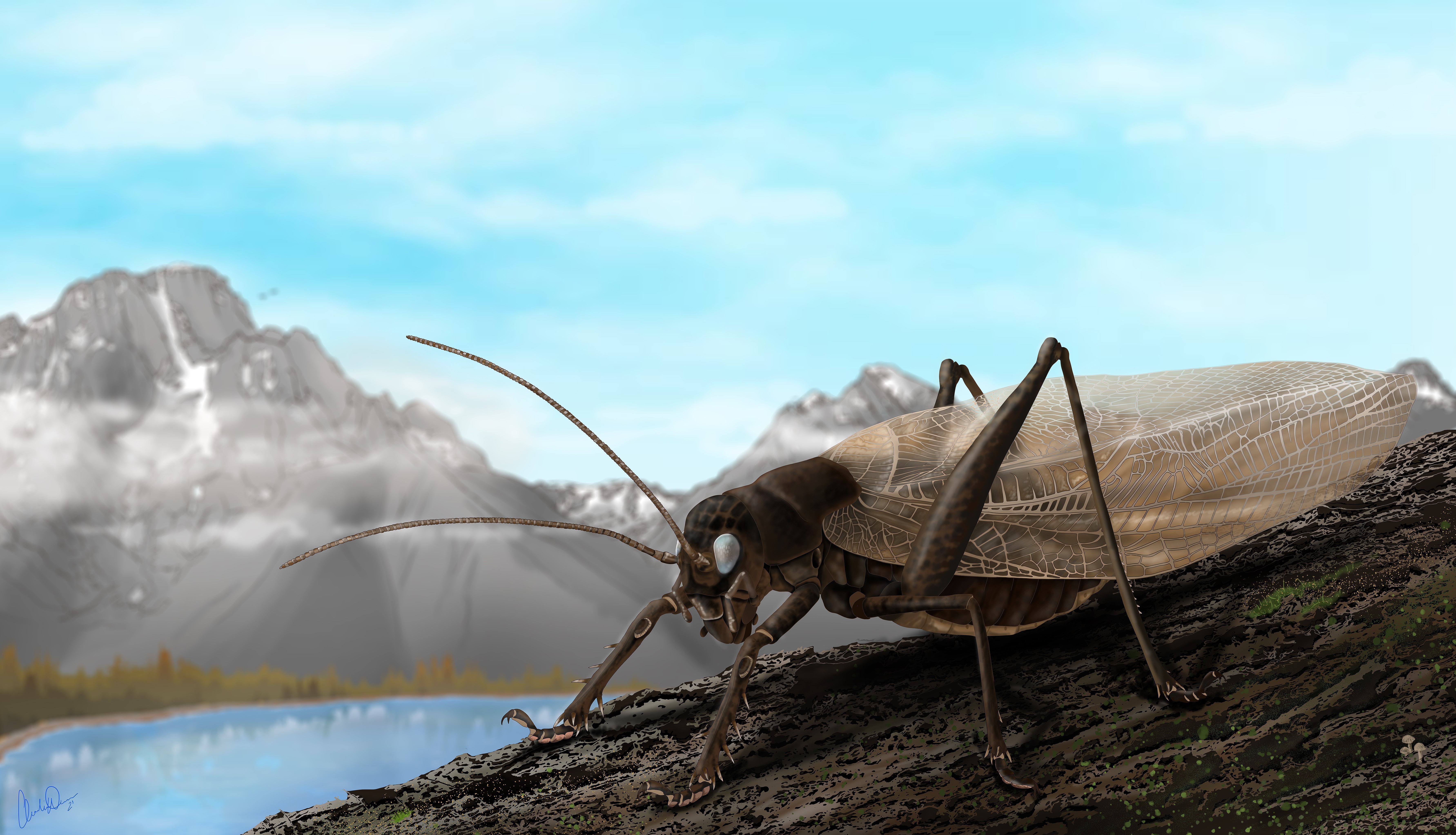

Supplement: S1 Fig — Illustrated by CW. (JPG) [file pone.0270498.s003.jpg]
